# Phononic Fano resonances in graphene nanoribbons with local defects

**DOI:** 10.1038/s41598-017-04987-w

**Published:** 2017-07-05

**Authors:** Alexander V. Savin, Yuri S. Kivshar

**Affiliations:** 1 Semenov Institute of Chemical Physics, Russian Academy of Science, Moscow, 119991 Russia; 2 Nonlinear Physics Center, Australian National University, Canberra, ACT 2601 Australia; 3 ITMO University, St. Petersburg, 194021 Russia

## Abstract

We study the interaction between localized vibrational modes and propagating phonons in graphene nanoribbons with different types of localized internal and edge defects. We analyze discrete eigenmodes of the nanoribbons with defects and also employ direct numerical simulations of the ballistic phonon and heat transport. We observe a partial suppression of the phonon transport due to the so-called *phononic Fano resonances* originating from interference between localized and propagating phonons. We observe lower transmission for the defects which support larger number of localized eigenmodes. The Fano resonance is also manifested in the reduction of the heat transport along the graphene stripe, when each of the local defects reduces the amount of the heat flow transmitted through the nanoribbon, with the effect being more pronounced at low temperatures when the thermal energy transfer is dominated by the phonon transport. We also study the similar problems for edge defects in graphene nanoribbons and demonstrate that a reduction of the thermal conductivity is proportional to the length of a rough edge of the nanoribbon with edge defects.

## Introduction

The Fano resonance is widely known across many different branches of physics^1^. From the viewpoint of the fundamental physics, the Fano resonance may appear in systems characterized by a certain discrete energy state that interacts with the continuum spectrum through an interface effect. Usually, the discrete state is created by a defect that allows one (or several) additional propagation paths in the wave scattering which interact constructively or destructively. In the transmission line, this interference effect leads to either perfect transmission or perfect reflection, producing a sharp asymmetric profile. In a classical paper^2^, Ugo Fano derived the general formula which describes asymmetric line shape of the transmission or absorption lines:

1 $$F(\varepsilon )={(\varepsilon +f)}^{2}/({\varepsilon }^{2}+\mathrm{1),}$$

where *ε* = (*E* − *E*_*R*_)/(Γ/2) is the dimensionless energy in units of the resonance width Γ, *f* is the asymmetry parameter (Fano factor), and *E*_*R*_ is the resonance energy.

One of the simplest models that can describe the resonant coupling and interaction between a discrete state and continuum spectrum is the so-called Fano-Anderson model^3, 4^, which describes a linear “atomic” chain with the nearest-neighbor interaction forces and interacting with a defect state through the nearest neighbors. This simple model allows one to describe the basics physics of the Fano resonance in a simple way.

**Figure 1:**
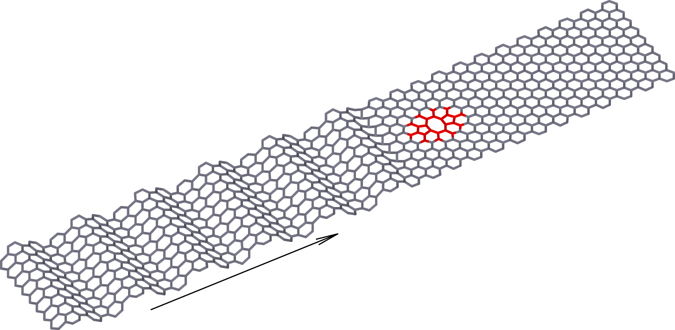
Schematic of the scattering of a longitudinal monochromatic wave by a double-vacancy V_2_(585) defect in an armchair graphene nanoribbon. Region of the defect is marked by a color. An arrow shows the direction of the wave propagation.

In this paper we reveal that the effective Fano-Anderson discrete model can be realized in the problem of phonons propagating in carbon nanoribbons with local structured defects (see an example in Fig. 1). Here an effective array is formed by structural elements (elementary unit cells) of the nanoribbon, and the defect states appear due to the localized vibrational modes supported by structural defects. In particular, we demonstrate the resonant coupling and interaction between localized modes supported by defects and the propagating phonons of the nanoribbon manifested through the phononic Fano resonances.

It is well known that the presence of defects can modify the properties of graphene. In particular, local defects can change the absorption properties of graphene^5^, spatially extended defects allow creating nanoengineering defect structures^6^, and linear arrays of defects can change the graphene conductivity^7–10^. Various types of defects in graphene can be divided into two large classes: local (or point) defects and linear defects^11^. Point defects in graphene act as scattering centers for electron and phonon waves^12–14^. In contrast, linear defects are responsible for static deformations of stretched nanoribbons. In this paper, we focus on the effects produced by local effects in the phonon scattering.

Rigidity and perfect local order of carbon nanostructures make them ideal nanoscale waveguides for a transport of phonons and thermal energy over long distances. This is observed in many experiments on the study of heat transport as an anomalously high thermal conductivity of isolated carbon nanotubes^15–17^ and graphene nanoribbons^18–20^.

Here we study the effect of localized modes supported by structural defects in graphene nanoribbons on the phonon transport and thermal conductivity. We consider several, most commonly discussed types of internal and edge defects in a nanoribbon and demonstrate that each of such defects support a number of localized vibrational eigenmodes. Being excited in the nanoribbon, such localized modes interact with propagating phonons and partially reflect them, thus modifying the thermal conductivity. Classification of defects and their characteristics can be found in the review paper^11^.

**Figure 2:**
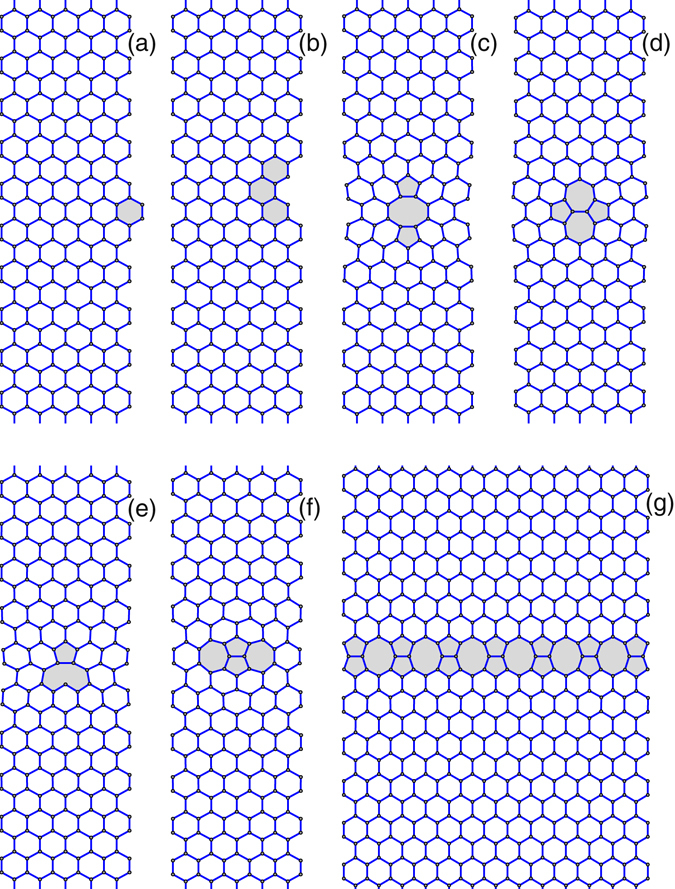
Localized defects in armchair graphene nanoribbon with the width *D* = 1.229 nm: (**a**) concave edge defect ED_+2_ (two additional carbon atoms); (**b**) convex edge defect ED_−2_ (two carbon atoms missing); (**c**) double vacancy V_2_(585); (**d**) Stone-Wales defect SW(5577); (e) single vacancy V_1_(59); (**f**) inverse Stone-Wales defect I_2_(7557). (**g**) Nanoribbon of the width *D* = 3.192 nm with an transverse array of the inverse Stone-Walles defects. Gray color marks a change of the lattice structure introduced by the presence of a defect.

**Figure 3:**
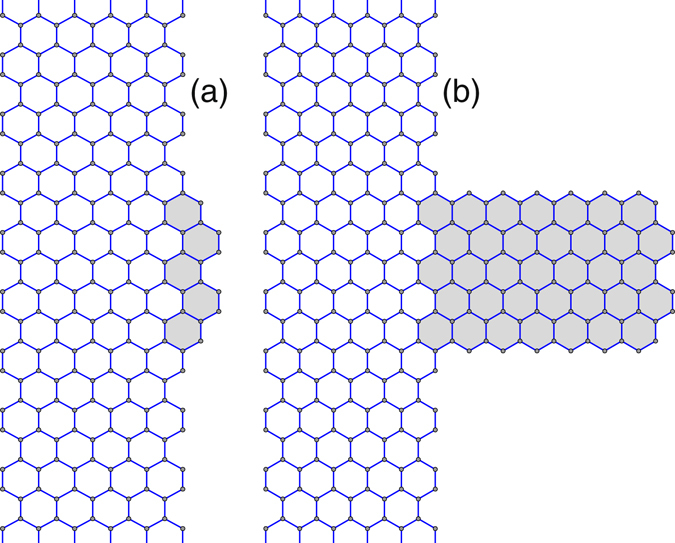
Example of an armchair graphene nanoribbon with an edge pillar defect: a small piece of a carbon structure is attached perpendicular to the nanoribbon. Width of the main nanoribbon is *D*_*a*_ = 1.228 nm, width of the zigzag nanoribbon is *D*_*z*_ = 1.134 nm, length of the edge pillar is (**a**) *L*_*p*_ = 0.246 nm and (**b**) *L*_*p*_ = 1.719 nm. Gray color marks a change of the lattice structure of the ideal armchair nanoribbon.

**Figure 4:**
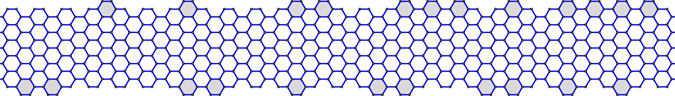
Example of an armchair carbon nanoribbon with rough edges in its central part. Edge defects creating the rough edges are marked by grey. Width of the nanoribbon *D*_*a*_ = 1.228 nm, density of defects *p*_*d*_ = 0.5.

We study several model problems for armchair graphene nanoribbons (shown in Figs 1 and 2) to demonstrate the effects produced by interaction of localized vibrational modes with propagating phonons. We consider six types of local defects placed at the center of the graphene nanoribbon, an extended defect in the form of an array of defects placed across the nanoribbon width (see Fig. 2), and different types of edge defects (see Figs 3 and 4). We demonstrate that each local defect supports a finite number of localized oscillatory states, and the number of modes is larger for narrow nanoribbons. We reveal that such localized modes may reduce or even reflect propagating phonons through the mechanism of Fano resonances, thus reducing the value of the transmission coefficient and subsequently the phonon transport and thermal conductivity. The Fano resonance is also found to reduce the heat transport along the graphene nanoribbon, with the effect being more profound at low temperature when the energy transfer is dominated by the phonon transport.

Extended defects support larger number of localized modes, and they should scatter phonons more intensively. A rough edge of the graphene nanoribbon represents one of such extended defects, and our results demonstrate that the value of thermal conductivity decreases monotonically with the length of the nanoribbon’s rough-edge region.

## Results

To get a deeper insight into the physics of resonant scattering observed in our rather complex system, first we consider the so-called Fano-Anderson model^3^ which is the simplest model describing the physics of the Fano resonance in discrete chains. More specifically, we consider a modified model describes by the following Hamiltonian^4^

2 $$H=\sum _{n}(E{|{\varphi }_{n}|}^{2}+C{\varphi }_{n}{\varphi }_{n-1}^{\ast })+{E}_{d}{|\phi |}^{2}+\sum _{j=-{n}_{d}}^{{n}_{d}}{V}_{j}{\phi }^{\ast }{\varphi }_{j}+\,{\rm{c}}{\rm{.c}}{\rm{.}}\,,$$

where the asterisk denotes complex conjugations and all coefficients are real numbers. This model describes the interaction of two subsystems. One subsystem consists of a straight linear chains with complex field amplitude *ϕ*_*n*_ at site *n* with local energy value *E* and which are coupled by nearest-neighbor coupling *C*. The second subsystem consist of an additional discrete state *ϕ* with local energy value *E*_*d*_. The interaction between these two subsystems is described by the coupling coefficients *V*_*j*_ (2*n*_*d*_ + 1 is the number of couplings between two subsystems). From the lattice Hamiltonian, we derive a system of coupled linear dynamic equations

$$i{\dot{\varphi }}_{n}=E{\varphi }_{n}+C({\varphi }_{n-1}+{\varphi }_{n+1})+\phi \sum _{j=-{n}_{d}}^{{n}_{d}}{V}_{j}{\delta }_{nj},$$

3 $$i\dot{\phi }={E}_{d}\phi +\sum _{j=-{n}_{d}}^{{n}_{d}}{V}_{j}{\varphi }_{j}\mathrm{.}$$

For further analysis, we look for stationary solutions of this system in the form

4 $${\varphi }_{n}(t)={A}_{n}\exp (-i\omega t),\quad \phi (t)=B\exp (-i\omega t),$$

which allows us to describe elastic scattering processes by means of a system of nonlinear algebraic equations

5 $$\begin{array}{rcl}(\omega -E){A}_{n} & = & C({A}_{n-1}+{A}_{n+1})+B\sum _{j=-{n}_{d}}^{{n}_{d}}{V}_{j}{\delta }_{nj},\\ (\omega -{E}_{d})B & = & \sum _{j=-{n}_{d}}^{{n}_{d}}{V}_{j}{A}_{j}\mathrm{.}\end{array}$$

We consider the scattering of plane waves *ϕ*_*n*_(*t*) = *Ae* ^*i*(*qn*−*ωt*)^, described by the wave number *q* ∈ [0, *π*] and dispersion $\omega (q)=E+2C\,\cos (q)$. Using the second equation of the system (5), we find a simple link between two defect-site characteristic,

6 $$B=\sum _{j=-{n}_{d}}^{{n}_{d}}{V}_{j}{A}_{j}/(\omega -{E}_{d}),$$

and obtain a single equation with a one-site scattering potential

7 $$(\omega -E){A}_{n}=C({A}_{n-1}+{A}_{n+1})+\sum _{j=-{n}_{d}}^{{n}_{d}}{c}_{n}{V}_{n}{V}_{j}{A}_{j}/(\omega -{E}_{d}),$$

where coefficient *c*_*n*_ = 0 for |*n*| > *n*_*d*_ and *c*_*n*_ = 1 for |*n*| ≤ *n*_*d*_.

For the scattering problem, we consider the boundary conditions

8 $${A}_{n}=\{\begin{array}{ll}{a}_{i}\exp (iqn)+{a}_{r}\exp (-iqn), & n\mathrm{ < 0,}\\ {a}_{t}\exp (iqn), & n\mathrm{ > 0,}\end{array}$$

where *a*_*i*_, *a*_*r*_, and *a*_*t*_ are incoming, reflected, and transmitted wave amplitudes far from the defect site. According to Eq. (7), the strength of the scattering potential depends on the incoming frequency *ω*, and the system should demonstrate *resonant scattering*. If the frequency of the defect is placed in the propagation frequency band – i.e., *E*_*d*_ ∈ [*E*, *E* + 2*C*] – the scattering potential in Eq. (7) becomes infinitely large at *ω* = *E*_*d*_, and this will lead to total reflection of the incoming wave.

In the simple case of local coupling in the chain, the transmission coefficient *T* = |*a*_*t*_|^2^/|*a*_*i*_|^2^ can be easily calculated since *n*_*d*_ = 0. In this case, the equation (7) is reduced to

9 $$(\omega -E){A}_{n}=C({A}_{n-1}+{A}_{n+1})+{V}_{0}^{2}{A}_{n}{\delta }_{n0}/(\omega -{E}_{d}),$$

and the transmission coefficient^4^

10 $$T={\alpha }^{2}(q)/[{\alpha }^{2}(q)+\mathrm{1],}$$

where $$\alpha (q)=2C(\omega (q)-{E}_{d})\sin (q)/{V}_{0}^{2}$$.

A graphene nanoribbon has *N*_*e*_ atoms in the elementary cell so its dispersion curve consists of 3*N*_*e*_ branches marked as ${\{{\omega }_{i}(q)\}}_{i=1}^{3{N}_{e}}$. One-dimensional chain discussed above is suitable for qualitative modeling of one of such branches (and respective modes) characterized by the frequency dependence *ω*_*i*_(*q*), so that *ϕ*_*n*_ is an effective wave function of the mode excited in the *n*-th elementary cell, and *ϕ* is the amplitude of the localized mode at a defect with the frequency *ω*_*d*_ = *E*_*d*_.

Our study of a simplifies model suggests that localized mode supported by a defect can interact with the propagating modes of the nanoribbon with the resonant reflection and reduction of the transmission coefficient. This is expected to occur if the frequency of the localized mode lies in the frequency band of the extended phonon modes, namely ${{\rm{\min }}}_{q}{\omega }_{i}(q)\le {\omega }_{d}\le {{\rm{\max }}}_{q}{\omega }_{i}(q)$, so propagating phonons with this frequency will be completely reflected by the defect, and the transmission coefficient vanishes in this point, namely *T*(*ω*_*d*_) = 0. We confirm this result below with the use of the complete model of the nanoribbon.

### Transmission coefficient for nanoribbons with defects

Each local defect supports several different localized oscillatory modes, the number of such modes depends on the width of the nanoribbon, and it is larger for narrow nanoribbons. For wider nanoribbons, the number of localized modes coincide with the modes of an infinite graphene sheet^21^. For the scattering problem, we expect that such local oscillatory states will interact with propagating phonons when their frequencies overlap, so the defect can suppress the phonon transmission or even reflect phonons completely.

The analysis of the eigenfrequencies and eigenmodes of the graphene nanoribbon with a defect embedded in its center allows not only find the localized modes but also calculating the transmission coefficient for phonons for each branch of the dispersion curve.

Let us consider the propagation of a photon wavepacket in the nanoribbon with a localized defect. We take the nanoribbon composed of *N* = 203 elementary cells with a defect placed in the cell with the number *N*_0_ = (*N* − 1)/2. We assume that the wavepacket is characterized by the dimensionless wave number *q* (where 0 < *q* < *π*), the frequency *ω*(*q*) and the eigenvector **v** = **v**_*R*_ + *i* **v**_*I*_ (**v**_*R*_ and **v**_*I*_ are the real and imaginary parts, respectively) that is a solution of the eigenvalue problem (25). For describing the wavepacket, we consider the initial conditions

11 $$\begin{array}{c}{{\bf{x}}}_{n}(0)={A}_{n}[{{\bf{v}}}_{R}\,\cos (qn)-{{\bf{v}}}_{I}\,\sin (qn)],\\ {{\bf{x}}}_{n}(0)=\omega {A}_{n}[{{\bf{v}}}_{R}\,\sin (qn)+{{\bf{v}}}_{I}\,\cos (qn)],\end{array}$$

where the vector **x**_*n*_ describes the displacements of atoms in the *n*-th elementary cell. Localized amplitudes *A*_*n*_ define the shape of the wavepacket, that we select in the form,

$$\begin{array}{c}{A}_{n}={A}_{0}/\,\cosh \,[\mu (n-{N}_{1})],\quad {\rm{for}}\quad n < {N}_{0},\\ {A}_{n}=\mathrm{0,}\quad {\rm{for}}\,\quad n\ge {N}_{0},\end{array}$$

where the number *N*_1_ = *N*/4 provides the initial location of the wavepacket, the parameter *μ* = 0.1 characterizes its extension, and *A*_0_ is the initial amplitude (not important for the study of the linear propagation).

Dynamics of harmonic oscillations of the nanoribbon is described by the system of linear equations (28) with a symmetric matrix of the second-order derivatives (29) of the size 3*N*_*a*_ × 3*N*_*a*_, where *N*_*a*_ is the number of atoms in the nanoribbon. Dimension of this matrix can be reduced provided one separate in-plane and out-of-plane vibrations.

The linear system of the motion equations (28) can be written in the form

12 $$-\ddot{y}={{\bf{M}}}^{-\mathrm{1/2}}{\bf{B}}{{\bf{M}}}^{-\mathrm{1/2}}{\bf{y}},$$

where the vector ${\bf{y}}={\{{{\bf{y}}}_{n}\}}_{n=1}^{N}$, ${{\bf{y}}}_{n}={{\bf{M}}}_{{\bf{n}}}^{-\mathrm{1/2}}{{\bf{x}}}_{n}$ (*M*_*n*_ is the diagonal matrix masses of the *n*-th elementary cell of the nanoribbon). We define ${\{{\omega }_{j}^{2},{{\bf{e}}}_{j}\}}_{j=1}^{3{N}_{a}}$ as the eigenvalue and eigenfunction of the eigenvalue problem (31), so that the general solution of the linear system (12) can be presented in the form,

13 $$y(t)=\sum _{j=1}^{3{N}_{a}}\,[{C}_{j\mathrm{,1}}\,\cos ({\omega }_{j}t)+{C}_{j\mathrm{,2}}\,\sin ({\omega }_{j}t)]{{\bf{e}}}_{j},$$

To take into account the initial condition (11), in the sum (13) we should specify the coefficients,

$${C}_{j\mathrm{,1}}=({{\bf{M}}}^{-\mathrm{1/2}}{\bf{x}}\mathrm{(0),}{{\bf{e}}}_{j}),\,\,\,{C}_{j\mathrm{,2}}=({{\bf{M}}}^{-\mathrm{1/2}}\dot{{\bf{x}}}\mathrm{(0),}{{\bf{e}}}_{j})/{\omega }_{j},$$

where the vectors are: ${\bf{x}}(0)={\{{{\bf{x}}}_{n}(0)\}}_{n=1}^{N}$, ${\bf{x}}(0)={\{{\dot{{\bf{x}}}}_{n}(0)\}}_{n=1}^{N}$.

We notice that for modeling the wavepacket dynamics we do not need integrating numerically the system of the motion equations, since it is sufficient to analyze the properties of the exact solution (13).

**Figure 5:**
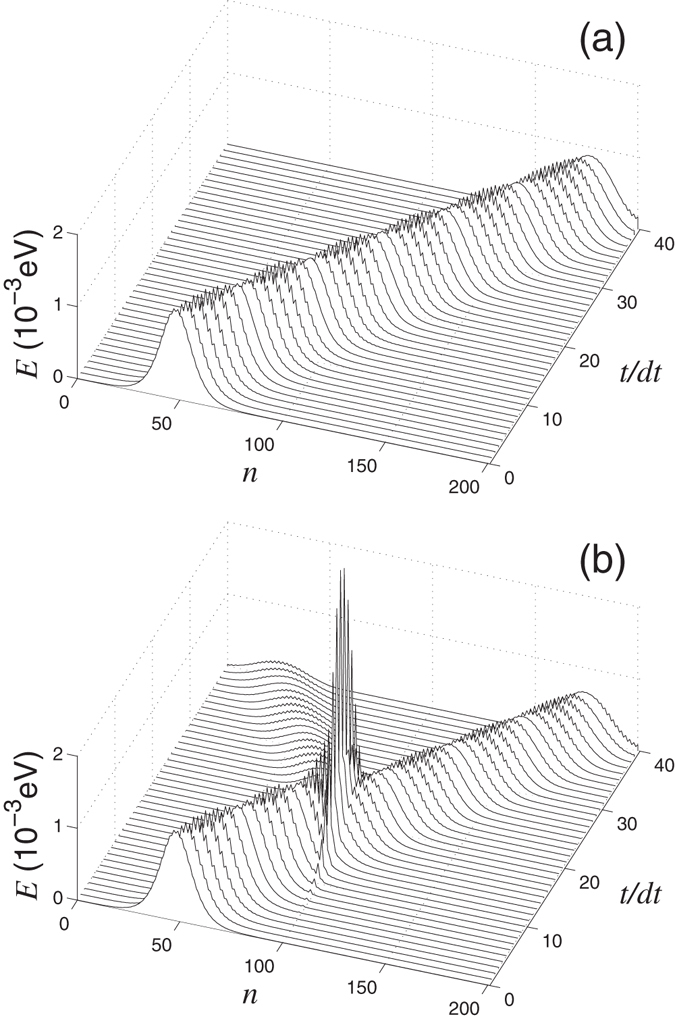
Propagation of a wavepacket of the out-of-plane phonons in (**a**) ideal nanoribbon and (**b**) nanoribbon with the double vacancy V_2_(585) (nanoribbon width *D* = 1.23 nm) for wave number *q* = 0.5*π*, frequency *ω*(*q*) = 880.9 cm^−1^ (the wavepacket amplitude *A* = 0.0003 Å). Shown is the energy distribution at different time values *t* along the nanoribbon (*n* is the number of the elementary unit cell), time step *dt* = 0.2 ps. Transmission coefficient *T*(*ω*, *q*) = 0.7694.

**Figure 6:**
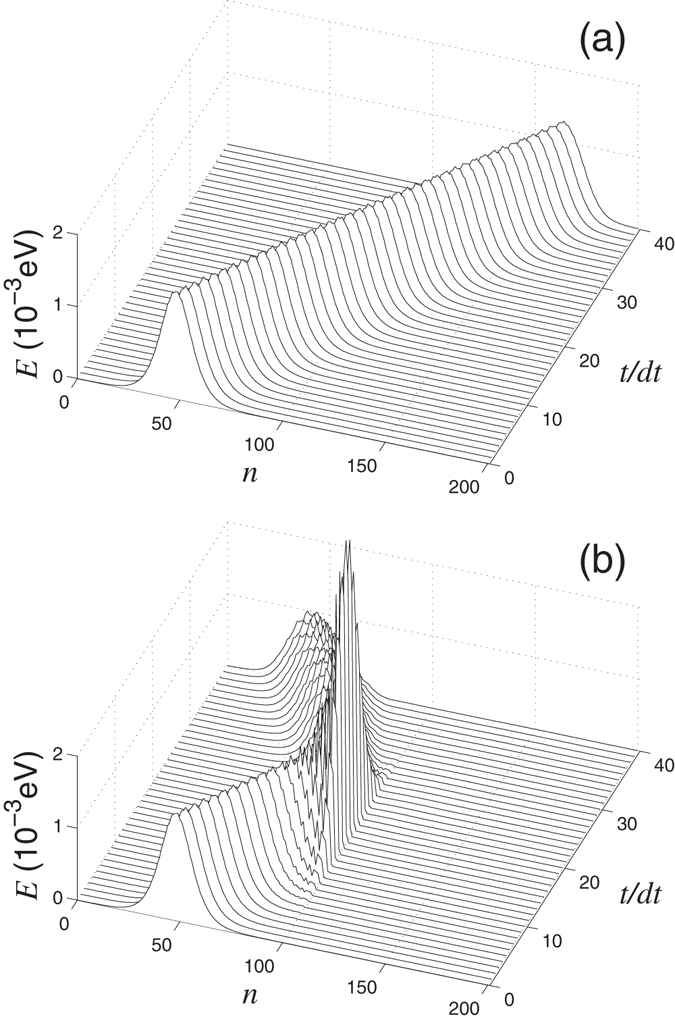
Propagation of a wavepacket of the in-plane phonons in (**a**) ideal nanoribbon and (**b**) nanoribbon with the double vacancy V_2_(585) (nanoribbon width *D* = 1.23 nm) for wave number *q* = 0.6*π*, frequency *ω*(*q*) = 1569.3 cm^−1^ (the wavepacket amplitude *A* = 0.0003 Å). Shown is the energy distribution at different time values *t* along the nanoribbon (*n* is the number of the elementary unit cell), time step *dt* = 0.15 ps. Transmission coefficient *T*(*ω*, *q*) = 0.0042.

In an ideal nanoribbon, the wavepacket will propagate with the group velocity *s* = *adω*/*dq*. For the period of time *t* = *Na*/2*s*, the main part of the wavepacket will be found on the right part of the nanoribbon, see Figs 5(a) and 6(a). However, when a defect is placed at the center of the nanoribbon, the wavepacket can pass though the defect, or get reflected from the defect partially or completely, see Figs 5(b) and 6(b). The transmission coefficient can be calculated directly, as a ratio of the energy of the wavepacket in the right side of the nanoribbon with a defect to the energy of the same wavepacket in the nanoribbon without defects, namely

$$T(\omega ,q)={E}_{r\mathrm{,1}}(t)/{E}_{r\mathrm{,0}}(t),\quad {\rm{where}}\quad {E}_{r,i}=\sum _{n={N}_{1}+2}^{N}{E}_{n}(t),$$

${\{{E}_{n}(t)\}}_{n=1}^{N}$ is the distribution the oscillation energy through the elementary cells of the nanoribbon with (*i* = 1) and without (*i* = 0) defects.

**Figure 7:**
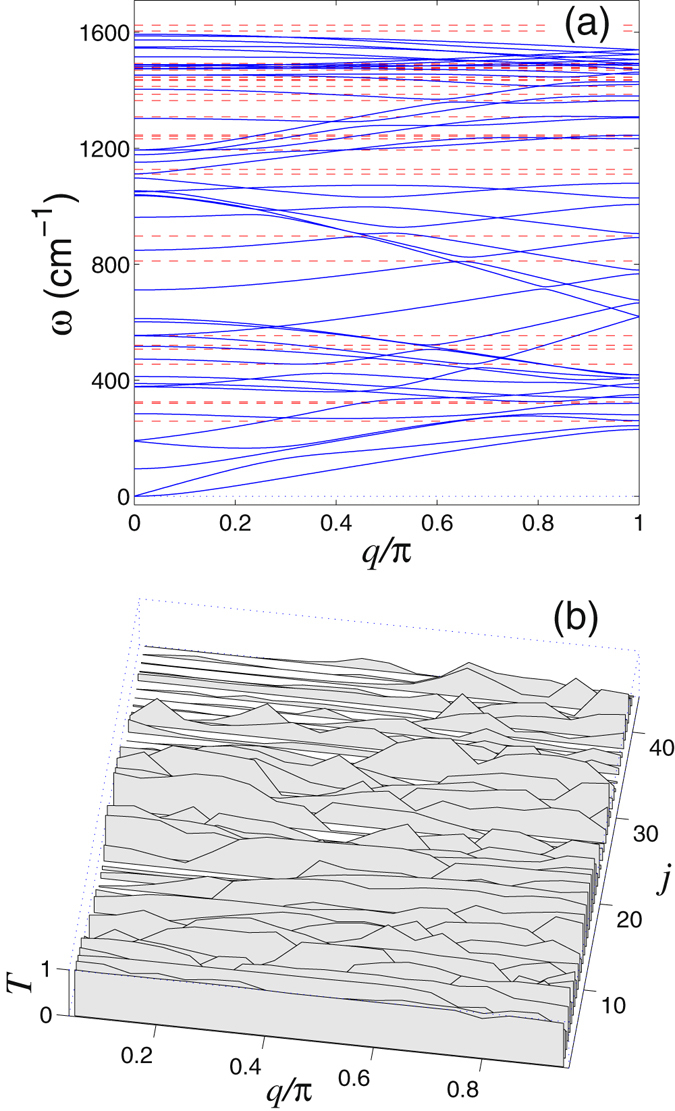
(**a**) Dispersion curves (2*N*_*e*_ in total) of the in-plane oscillations of an armchair nanoribbon. Number of atoms in the elementary cell is *N*_*e*_ = 22. (**b**) Transmission coefficient *T* vs. wavenumber *q* for each dispersion curve (*j* stands for the sequential number of a dispersion curve) for phonons scattered by the double vacancy V_2_(585). The average transmission coefficient of such phonons is $\bar{T}=0.445$. Horizontal dashed straight lines mark the eigenfrequencies of the in-plane localized vibrations of the defect.

**Figure 8:**
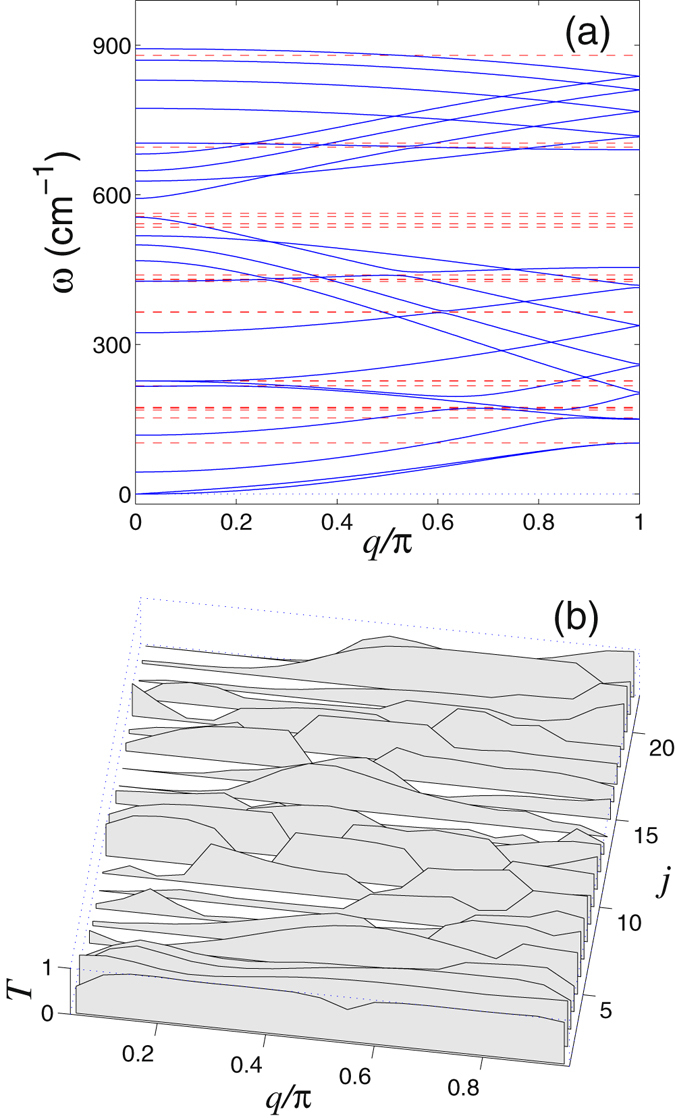
Dispersion curves (*N*_*e*_ in total) of the out-of-plane oscillations of an armchair nanoribbon. Number of atoms in the elementary cell is *N*_*e*_ = 22. (**b**) Transmission coefficient *T* vs. wavenumber *q* for each dispersion curve (*j* stands for the sequential number of a dispersion curve) for phonons scattered by the double vacancy V_2_(585). The average transmission coefficient of such phonons is $\bar{T}=0.533$. Horizontal dashed straight lines mark the eigenfrequencies out-of-plane localized vibrations of the defect.

Dependence of the transmission coefficient *T* on the phonon frequency *ω* and wave number *q* is shown in Figs 7 and 8. As follows from these results, the transmission coefficient of phonons decreases as soon as the dispersion curves approaches the frequency of the localized state supported by the defect. In this case we observe the Fano resonance when the phonon interact resonantly with the localized state, that may result in the partial or total reflection of the phonon wavepacket from the defect, see Figs 5 and 6.

Influence of defects on the phonon transport in the graphene nanoribbon can be characterized by the average transmission coefficient $\bar{T}$ calculated through the averaging for all dispersion curves and all value of the wave number. As an example, for the nanoribbon with the width *D* = 1.23 nm including the double vacancy V_2_(585), this averaged transmission coefficient can be calculated to be $\bar{T}=0.474$, meaning that in average at least a half of the incoming phonons get reflected.

Dependence of the average transmission coefficient of phonons $\bar{T}$ vs. the nanoribbon width *D* is summarized in Table 1. As follows from these data, the strongest suppression of the phonon transport is observed in nanoribbons with a transverse chain of inverse Stone-Wales defects. Among all point defects, the largest effect is produced by the double vacancy V_2_(585). Also, the phonon scattering is stronger for more narrow nanoribbons.

**Table 1:** Dependence of the average transmission coefficient of phonons $\bar{T}$ on the nanoribbon width *D* and a type of defect.

*D* (nm)	1.23	1.72	2.21	3.19
without defect	1	1	1	1
ED_+2_	0.663	0.725	0.760	0.808
ED_−2_	0.685	0.744	0.778	0.822
V_2_(585)	0.474	0.541	0.603	0.678
SW(5577)	0.528	0.576	0.634	0.700
V_1_(59)	0.483	0.564	0.629	0.707
I_2_(7557)	0.529	0.590	0.659	0.731
I_2_ chain	0.461	0.481	0.510	0.546

We notice that the value of the average transmission coefficient $\bar{T}$ can not characterize precisely the suppression of the phonon transport because it does not take into account the group velocities of phonons. The most relevant analysis we conduct below is associated with the study of thermal conductivity in the nanoribbons with defects and its comparison with thermal conductivity in an ideal nanoribbon.

### Effect of internal defects on thermal energy transfer

In order to model the thermal transport along the nanoribbon, we place its edges with *N*_0_ = 12 edge elementary unit cells into the thermostats with the temperatures *T*_+_ (left edge) and *T*_−_ (right edge) so that *T*_+_ > *T*_−_, and then calculate the temperature gradient along the nanoribbon and continuous heat flow from the warmer to colder end of the nanoribbon.

We define the 3*N*_*e*_-dimensional coordinate vector ${{\bf{x}}}_{n}={\{{{\bf{u}}}_{(n,k)}\}}_{k=1}^{{N}_{e}}$ which determines the atom coordinates of an elementary cell *n*, and then write Hamiltonian (17) in the form

14 $$H=\sum _{n}{h}_{n}=\sum _{n}[\frac{1}{2}({\bf{M}}{\dot{{\bf{x}}}}_{n},{\dot{{\bf{x}}}}_{n})+P({{\bf{x}}}_{n-1},{{\bf{x}}}_{n},{{\bf{x}}}_{n+1})],$$

where the first term (a scalar product of two vectors) describes the kinetic energy of the atoms (*M* is diagonal mass matrix of the *n*th elementary cell), and second term (a function of three variables) describes the interaction between the atoms in the cell and with the atoms of neighboring cells.

Hamiltonian (14) generates the systems of equations of motion,

15 $$-{\bf{M}}{\ddot{{\bf{x}}}}_{n}={{\bf{F}}}_{n}={{\bf{P}}}_{\mathrm{1,}n+1}+{{\bf{P}}}_{\mathrm{2,}n}+{{\bf{P}}}_{\mathrm{3,}n-1},$$

where the function **P**_*i*,*n*_ = **P**_*i*_(**x**_*n*−1_, **x**_*n*_, **x**_*n*+1_), **P**_*i*_ = ∂*P*(**x**_1_, **x**_2_, **x**_3_)/∂**x**_*i*_, *i* = 1, 2, 3.

Local heat flux through the *n*th elementary cell, *j*_*n*_ determines a local longitudinal energy density *h*_*n*_ by means of a discrete continuity equation, ${\dot{h}}_{n}={j}_{n}-{j}_{n-1}$. Using the energy density from Eq. (14) and the motion equations [Eq. (15)], we obtain the general expression for the energy flux through the *n*th cross section of the nanoribbon,

$${j}_{n}=({{\bf{P}}}_{\mathrm{1,}n},{\dot{{\bf{x}}}}_{n-1})-({{\bf{P}}}_{\mathrm{3,}n-1},{\dot{{\bf{x}}}}_{n}\mathrm{).}$$

For a direct modeling of the heat transfer along the nanoribbon, we consider a nanoribbon of a fixed length (*N* − 1)*a* with fixed ends (period *a* = 3*ρ* = 4.254 Å is length of elementary cell). We place the first *N*_0_ = 12 segments into the Langevin thermostat at temperature *T*_+_, and the last *N*_0_ elementary cells, into the thermostat at *T*_−_. As a result, for modeling of the thermal conductivity we need integrating numerically the following system of equations:

16 $$\begin{array}{c}{\bf{M}}{\ddot{{\bf{x}}}}_{n}=-{{\bf{F}}}_{n}-{\rm{\Gamma }}{\bf{M}}{\dot{{\bf{x}}}}_{n}+{{\rm{\Xi }}}_{n}^{+},\,n=1,\ldots ,{N}_{0},\\ {\bf{M}}{\ddot{{\bf{x}}}}_{n}=-{{\bf{F}}}_{n},n={N}_{0},\ldots ,N-{N}_{0},\\ {\bf{M}}{\ddot{{\bf{x}}}}_{n}=-{{\bf{F}}}_{n}-{\rm{\Gamma }}{\bf{M}}{\dot{{\bf{x}}}}_{n}+{{\rm{\Xi }}}_{n}^{-},n=N-{N}_{0}+1,\ldots ,N,\end{array}$$

where Γ = 1/*t*_*r*_ is the damping coefficient (relaxation time *t*_*r*_ = 0.4 ps), and

$${{\rm{\Xi }}}_{n}^{\pm }={\{({\xi }_{n,k\mathrm{,1}}^{\pm },{\xi }_{n,k\mathrm{,2}}^{\pm },{\xi }_{n,k\mathrm{,3}}^{\pm })\}}_{k=1}^{{N}_{e}}$$

is 3*N*_*e*_-dimensional vector of normally distributed random forces normalized by conditions

$$\langle {\xi }_{n,k,i}^{\pm }({t}_{1}){\xi }_{m,l,j}^{\pm }({t}_{2})\rangle =2M\Gamma {k}_{B}{T}_{\pm }{\delta }_{nm}{\delta }_{kl}{\delta }_{ij}\delta ({t}_{1}-{t}_{2}).$$

We select the initial conditions for system [Eq. (16)] corresponding to the ground state of the nanoribbon, and solve the equations of motion numerically tracing the transition to the regime with a stationary heat flux. At the inner part of the nanoribbon *N*_0_ < *n* ≤ *N* − *N*_0_, we observe the formation of a temperature gradient corresponding to a constant flux. Distribution of the average values of temperature and heat flux along the nanoribbon can be found in the form

$${T}_{n}=\mathop{\mathrm{lim}}\limits_{t\to \infty }\frac{1}{3{N}_{e}{k}_{B}t}{\int }_{0}^{t}({\bf{M}}{\dot{{\bf{x}}}}_{n}(\tau ),{\dot{{\bf{x}}}}_{n}(\tau ))d\tau ,\quad {J}_{n}=\mathop{\mathrm{lim}}\limits_{t\to \infty }\frac{1}{t}{\int }_{0}^{t}a{j}_{n}(\tau )d\tau ,$$

where *k*_*B*_ is the Boltzmann constant.

**Figure 9:**
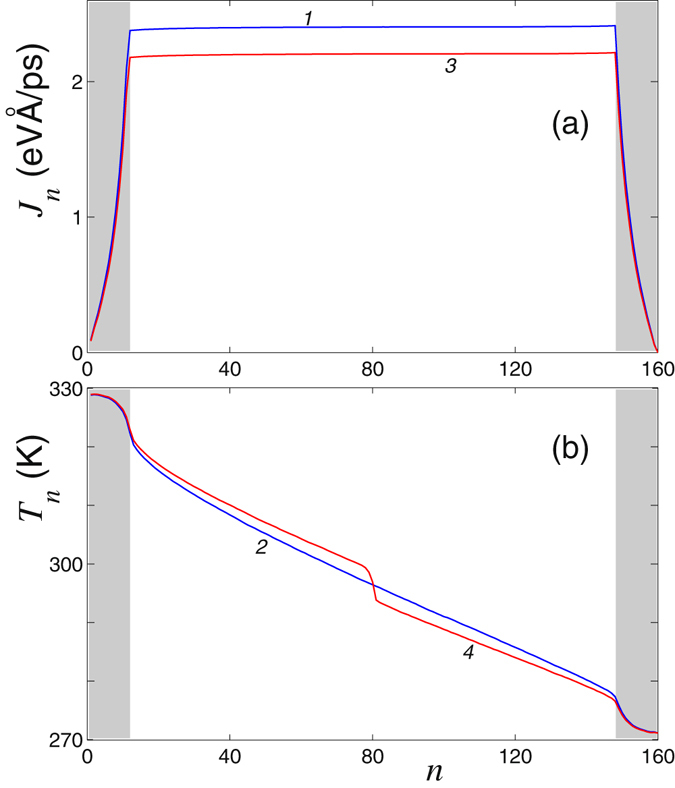
Spatial distribution of (**a**) local heat flux *J*_*n*_ and (**b**) temperature *T*_*n*_ along the nanoribbon with and without point defect. The nanoribbon width is *D* = 1.72 nm, and its length is *L* = 68 nm (*N* = 160). Thermostat temperature from the left end of the nanoribbon is *T*_+_  = 330 K, and from the right – *T*_−_ = 270 K. Grey color marks the end regions corresponding to the interaction of the nanoribbon with thermostat. Curves 1 and 2 correspond to the ideal nanoribbon, and curves 3 and 4 correspond to the nanoribbon with the point defect V_2_(585).

Distribution of the temperature and local heat flux along the nanoribbon is shown in Fig. 9. The heat flux in each cross section of the inner part of the nanoribbon should remain constant, namely, *J*_*n*_ ≡ *J* for *N*_0_ < *n* ≤ *N* − *N*_0_. The requirement of independence of the heat flux *J*_*n*_ on a local position *n* is a good criterion for the accuracy of numerical simulations, as well as it may be used to determine the integration time for calculating the mean values of *J*_*n*_ and *T*_*n*_. As follows from the figure, the heat flux remains constant along the central inner part of the nanoribbon [see Fig. 9(a)].

Let us compare the thermal energy transfer in the nanoribbons with and without defects. In the ideal nanoribbon, the stationary heat flux is created with a liner temperature gradient at the center of the nanoribbon. This gradient is accompanied by a temperature jump when a defect is placed into the nanoribbon [see Fig. 9(b)]. Our numerical modeling of the thermal conductivity shows that a defect leads to a reduction of the thermal energy flux, *J*_*d*_ < *J*_0_, where *J*_0_ is the thermal energy flux in the ideal nanoribbon, and *J*_*d*_ is the similar value for the case of a defect. We may characterize this reduction by the coefficient *T*_*e*_ = *J*_*d*_/*J*_0_ < 1.

A linear temperature gradient can be used to define the local coefficient of thermal conductivity,

$$\kappa ({N}_{i})=(N-2{N}_{0})J/({T}_{{N}_{0}}-{T}_{N-{N}_{0}})S,$$

where *N*_*i*_ = *N* − 2*N*_0_ is the number of periods in the central part of the nanoribbon, *S* = 2(*D* + 2*r*_*C*_)*r*_*C*_ is the area of the nanoribbon cross section (*D* – nanoribbon width, *r*_*c*_ = 1.85 Å – van der Waals carbon radius).

**Figure 10:**
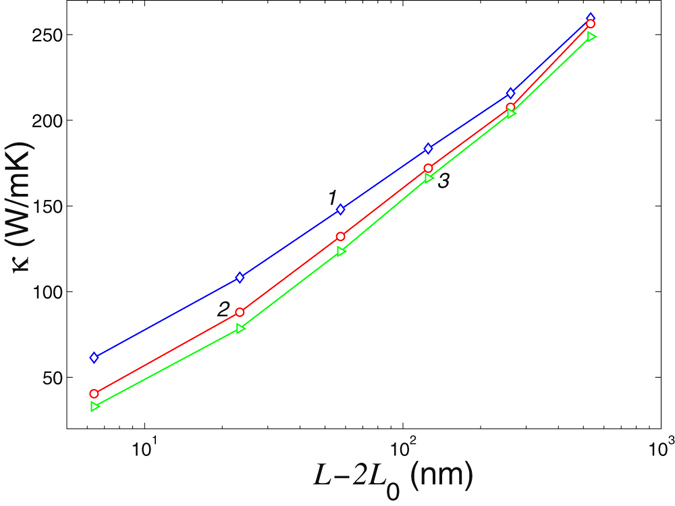
Coefficient of thermal conductivity *κ* vs. the length of the central part of the nanoribbon *L* − 2*L*_0_ for an ideal nanoribbon (curve 1), for a nanoribbon with a double vacancy V_2_(585) and a transverse array of the inverse Stone-Wales defects (curves 2 and 3). Nanoribbon width is *D* = 1.72 nm, temperature *T* = 300 *K*, *L*_0_ = *aN*_0_ is the length of the nanoribbon ends interacting with thermostat.

Dependence of the thermal conductivity of an ideal nanoribbon and a nanoribbon with a defect placed at its center is shown in Fig. 10. As follows from those results, the thermal conductivity of an ideal nanoribbon *κ* grows monotonously with the length *L* for *L* < 1 *μ*m. According to the earlier results^22^, the value of the thermal conductivity should saturate at the lengths *L* ~ 10^2^ ÷ 10^3^ *μ*m. The presence of a point defect leads to a reduction of the values of thermal conductivity for the small lengths *L* < 1 *μ*m. This reduction becomes smaller for longer lengths, and for *L* ≥ 1 *μ*m the influence of a local defect becomes negligible.

**Figure 11:**
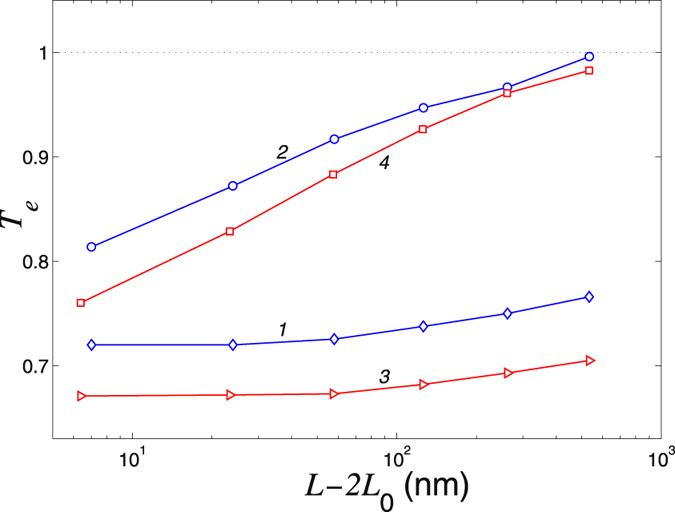
Reduction of the heat transport characterized by the coefficient *T*_*e*_, as a function of the nanoribbon length *L* in the nanoribbon with the point defect V_2_(585) (curves 1 and 2) and in the nanoribbon with a transverse linear chain of inverse Stone-Wales defects (curves 3 and 4). Curves 1 and 3 are for temperature *T* = 3 K, and the curves 2 and 4 are for temperature *T* = 300 K. Width of the nanoribbon is *D* = 1.72 nm.

To determine the reduction of the thermal flux and find the dependence of the coefficient *T*_*e*_(*T*) on temperature, we place the ends of the nanoribbon of the length *L*_0_ = 5 nm into thermostat with the temperatures *T*_±_ = (1 ± 0.1)*T* from the left and right sides, respectively. The characteristic dependence of the coefficient *T*_*e*_ on the length of the nanoribbon *L* is shown in Fig. 11. If the heat transport is conducted by noninteracting phonons, then the reduction coefficient *T*_*e*_ should not depend on the length *L*. This is indeed observed for low temperatures, so for *T* = 0.3K the coefficient *T*_*e*_ is almost independent on *L*, but for *T* = 3 K this dependence is observed for large lengths. However, for *T* = 300 K we observe a monotonous growth of *T*_*e*_ due to anharmonicity of the phonon scattering in the nanoribbon. In particular, for *L* = 544 nm the coefficient *T*_*e*_ riches the unity and thus for such long distances the influence of defects of the heat flow in the nanoribbon becomes negligible.

In the modeling of thermal conductivity for low temperatures, we can determine the reduction of the heat flow in the presence of defects *T*_*ph*_ due to the phonon energy transport. Dependence of *T*_*ph*_ on the width of the nanoribbon *D* and the type of local defect is summarized in Table 2. As follows from those results, the coefficient *T*_*ph*_ grows monotonically with the width of the nanoribbon, namely *T*_*ph*_ → 1 for *D* → ∞. However, the nanoribbon with a transverse chain of defects makes an exceptional case, here we always have *T*_*ph*_ < 0.7. Comparing these data with the data from Table 1 we come to the conclusion that ${T}_{ph} > \bar{T}$, and this is explained by the fact that the reduction of the phonon transport due to the presence of defects is associated with the scattering of slow phonons, whereas phonons with larger group velocities give a large contribution to the flow of heat energy.

**Table 2:** Reduction of the thermal conductivity characterized by the coefficient *T*_*ph*_ in the case of purely phonon transport along the nanoribbon.

*D* (nm)	1.23	1.72	2.21	3.19
without defect	1	1	1	1
ED_+2_	0.858	0.897	0.917	0.944
ED_−2_	0.835	0.882	0.905	0.938
V_2_(5-8-5)	0.650	0.720	0.767	0.829
SW(55–77	0.735	0.789	0.826	0.876
V_1_(5–9)	0.634	0.717	0.779	0.845
I_2_(7557)	0.725	0.792	0.836	0.887
I_2_ chain	0.654	0.672	0.684	0.697

Thus, each defect should lead to the reduction of the thermal conductivity. This reduction becomes substantial in for narrow nanoribbons, and especially for nanoribbons with rough surfaces^23^.

### Effect of edge defects on thermal energy transfer

Next, we evaluate the effect of edge defects on thermal conductivity of the nanoribbon. To do so, we carry out the modeling of the heat flow in an armchair graphene nanoribbon with an edge pillar defect, when a finite piece of a zigzag carbon structure is attached to the nanoribbon, as shown in Fig. 3.

For definiteness, we take an armchair nanoribbon with the width *D*_*a*_ = 1.228 nm and at its center we attach a side defect in the form of a zigzag nanoribbon of a finite width *D*_*z*_ = 1.134 nm and the length *L*_*p*_ that defines the length of the edge pillar defect [the smallest length of such a defect is *L*_*p*_ = 0.246 nm, see Fig. 3(a)].

Larger defects support a larger number of localized oscillatory modes. Therefore, defects with larger extension are likely to show stronger manifestation of Fano resonances, and stronger scattering effects, with decreasing thermal flow.

To model the heat flow, we place the ends of the nanoribbon of the length *L*_0_ = 5 nm into the Langevin thermostat with the temperatures *T*_±_ = (1 ± 0.1)*T* and study numerically the resulting distribution of the thermal energy flux along the nanoribbon. We compare the thermal conductivity of an ideal nanoribbon with the case of an edge pillar defect. Our results suggest that the surface defect leads always to a decrease of the the heat flow in the nanoribbon, *J*_*d*_ < *J*_0_, where *J*_0_ is the energy flow in the ideal nanoribbon and *J*_*d*_ is the energy flow in the nanoribbon with defects. The reduction coefficient is define as a ratio, *T*_*e*_ = *J*_*d*_/*J*_0_ < 1.

**Figure 12:**
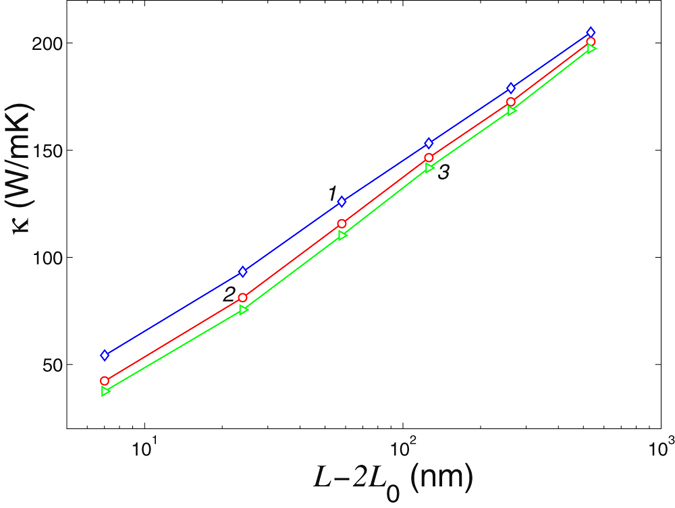
Coefficient of thermal conductivity *κ* vs. the length of the central part of the nanoribbon *L* − 2*L*_0_ for ideal nanoribbon (curve 1), for nanoribbon with an edge pilar with the lengths *L*_*p*_ = 0.245, 0.49 nm (curve 2, 3). Nanoribbon width is *D* = 1.228 nm, temperature *T* = 300 *K*.

Dependence of the thermal conductivity of an ideal nanoribbon of the width *D* = 1.228 nm and a nanoribbon with an edge pilar of different sizes is shown in Fig. 12. As follows from those results, the presence of a single edge defect leads to the substantial reduction of the thermal conductivity for the lengths *L* < 1 *μ*m. However, for larger lengths *L* ≥ 1 *μ*m such a defect has a very weak influence of the thermal conductivity. Nevertheless, a periodic array of such edge defects will have a substantial influence of thermal conductivity, similar to the studied case of cross-section modulated nanowires^24, 25^.

**Figure 13:**
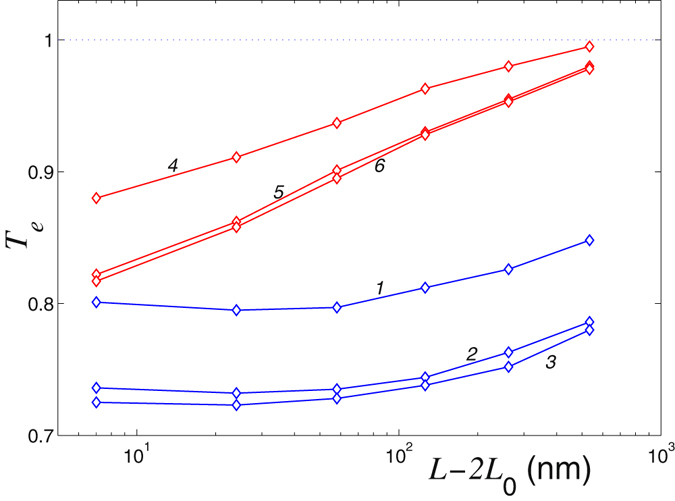
Reduction of the heat transport characterized by the coefficient *T*_*e*_, as a function of the nanoribbon length *L* in the nanoribbon with an edge pilar with the lengths *L*_*p*_ = 0.246, 0.49, 0.98 nm for temperature *T* = 3 K (curves 1, 2, 3) and *T* = 300 K (curves 4, 5, 6). Width of the nanoribbon is *D* = 1.228 nm.

The characteristic dependence of the coefficient *T*_*e*_ on the length *L* for different sizes of the edge pillar defects is shown in Fig. 13. For low temperature *T* = 3 K the reduction of the heat transport characterized by the coefficient *T*_*e*_ depends very weakly on the length of the nanoribbon, indicating primarily phonon mechanism of the energy transport. However, for *T* = 300 K the anharmonicity effects lead to a monotonous growth of the coefficient *T*_*e*_ with the nanoribbon length. For *L* > 700 nm the coefficient *T*_*e*_ approaches unity, i.e. for such long nanoribbon the presence of a defect does not change much the thermal conductivity.

The reduction of the heat flow depends also on the length of the defect *L*_*p*_. We notice that an increase of the length of the edge pilar reduces the transmission coefficient. However, we observe a saturation effect for *L*_*p*_ ≥ 1 nm when the transmission coefficient does not depend much on the length of the edge defect.

The presence of several edge defects enhance the phonon scattering resulting in a reduction of the thermal conductivity. A rough edge of the nanoribbon can be treated as a system of such edge defects place along the edge of the nanoribbon with some density *p*_*d*_, see Fig. 4. As a result, such a large number of defect should suppress substantially the heat transport in then nanoribbon reducing its thermal conductivity. Indeed, it is known that rough edges of the nanoribbon with the width *D*_*z*_ = 1.13 nm can suppress thermal conductivity by two orders of magnitude^23^.

In our current study, we demonstrate this effect by analyzing an armchair nanoribbon with rough edges. We take an ideal armchair nanoribbon with the width *D*_*a*_ = 1.228 nm and length *L*. Then we create rough edges in its center by adding, with the probability 0 ≤ *p*_*d*_ ≤ 1, two extra atoms of carbon to each four atoms of the edge by closing rings into hexagons, see Fig. 4. Each new hexagon create an edge defect, the density of these defect is defined by the probability *p*_*d*_ = 0.5. In such a model, in the limit *p*_*d*_ = 0, we have an ideal nanoribbon with the initial width *D*_*a*_ = 1.228 nm, but in the limit *p*_*d*_ = 1 the central part of the nanoribbon will a piece of an ideal wider nanoribbon with the width *D*_*a*_ = 1.474 nm.

To study thermal conductivity, we place the ends of the nanoribbon of the length *L*_0_ = 5 nm into a Langevin thermostat with the temperature *T*_±_ = (1 ± 0.1)*T* and determine the dependence of the thermal energy flow *J*_*d*_ in the nanoribbon with rough edges in its central part (with the density of edge defects *p*_*d*_ = 0.5) as a function of temperature *T* and the length of the nanoribbon *L*. We define the reduction of the thermal conductivity by the coefficient *T*_*e*_ = *J*_*d*_/*J*_0_, where *J*_0_ is the thermal flow in the nanoribbon without defects which we find independently in the case *p*_*d*_ = 0.

Results of our numerical studies are summarized in Table 3. As follows from these results, a growth of the section with rough edges leads to monotonous decrease of the coefficient *T*_*e*_ characterizing the relative reduction of the thermal energy flow. This suppression of thermal conductivity is more pronounced for low temperatures when the phonon scattering by edge defects is dominating.

**Table 3:** Dependence of thermal conductivity reduction *T*_*e*_ on the length of the rough edge of the nanoribbon *L* − 2*L*_0_ for two values of temperature *T* = 3 K and *T* = 300 K.

*L* − 2*L*_0_ (nm)	10.5	21.1	42.4	84.9
*T* = 3 K	0.503	0.377	0.257	0.166
*T* = 300 K	0.648	0.563	0.485	0.424

Width of the nanoribbon *D*_*a*_ = 1.228 nm, and the defect density *p*_*d*_ = 0.5.

## Discussion

By employed detailed numerical approach, we have studied the dynamics of localized vibrational modes supported by local and extended (line or surface) defects in graphene nanoribbons, as well as the influence of local defects on the phonon transport and thermal conductivity in the nanoribbons. We have observed the expected suppression of phonon scattering in the nanoribbons with defects, and we have reveal a link between this suppression and the phononic Fano resonances, manifested in partial or total resonant reflection of propagating phonons by localized modes excited at the defects.As a result, we have observed a reduction of the heat transport and thermal conductivity. The larger number of localized modes is supported by the defect, the stronger effect is observed.

We have studied several types of local defects in the nanoribbons and found that the strongest effect on the phonon transport is produced by double vacancies. The edge localized defects support a small number of localized modes, and their influence on the phonon transport is weaker. Stronger effects in the phonon scattering have been observed for larger T-stub edge pilar defects where the phonon energy flow is reduced with the growth of the transverse extension of the defect, with the maximum reduction observed for the width of 1 nm.

Extended defects are found to have a strong effect on the phonon scattering, since they scatter phonons more intensively due to larger number of localized vibrational mods they support. In particular, a rough edge of the graphene nanoribbon represents an extended defects, and our study has demonstrated that the thermal conductivity decays monotonically with the extension of the surface defect.

## Methods

**Figure 14:**
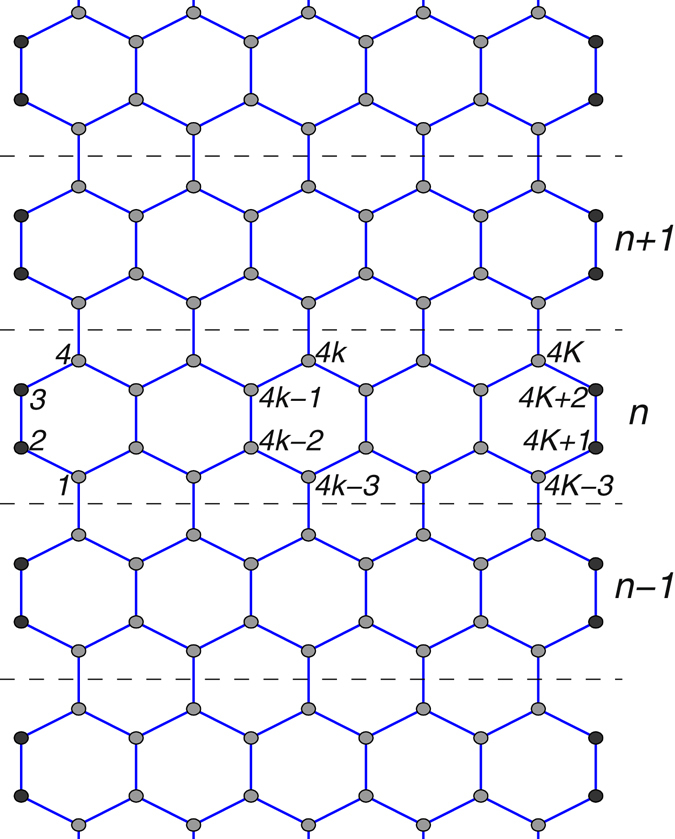
Schematic view of armchair nanoribbon with atom numbering. The edge atoms are shown as filled circles. Dotted lines separate the elementary cells of the nanoribbon. *N*_*e*_ = 4 *K* + 2 is the number of atoms in the elementary cell. Width of the nanoribbon *D* = 1.23 nm, the number of full hexagons in the cell *K* = 5.

To model numerically the dynamics of a graphene nanoribbon with local defects, we consider armchair nanoribbon with length *L* = 67.71 nm and width *D* = 1.23, 1.72, 2.21, 3.19 nm. The schematic structure of the nanoribbon and the atom numbering are shown in Fig. 14.

### Model

A finite-length nanoribbon consists of *N* elementary cells, each of them has *N*_*e*_ = 4 *K* + 2 carbon atoms, *N*_*h*_ = 2 *K* − 1 hexagons, where the number *K* = 2, 3, … In Fig. 14 we show edge atoms as filled circles. In a realistic case, the edge atoms are chemically modified. We consider hydrogen terminated graphene nanoribbon with the edge atoms corresponding to the group CH. In this study we take into account only a change of the effective mass of the edge atom: the mass of atoms inside the stripe is *M*_0_ = 12*m*_*p*_ and the large mass *M*_1_ = 13*m*_*p*_ for edge atoms (where *m*_*p*_ = 1.6603 × 10^−27^ kg is the proton mass).

**Figure 15:**
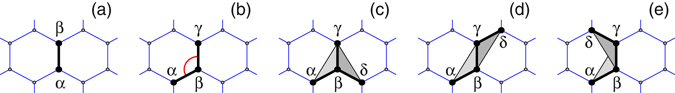
Configurations containing up to *i*th type of nearest-neighbor interactions for (**a**) *i* = 1, (**b**) *i* = 2, (**c**) *i* = 3, (**d**) *i* = 4, and (**e**) *i* = 5.

To describe nanoribbon oscillations, we start from the system Hamiltonian in the form

17 $$H=\sum _{n=1}^{N}\sum _{k=1}^{{N}_{e}}[\frac{1}{2}{M}_{(n,k)}({\dot{{\bf{u}}}}_{(n,k)},{\dot{{\bf{u}}}}_{(n,k)})+{P}_{(n,k)}],$$

where *M*_*α*_ is the mass of the carbon atom or carbon edge group with the index *α* = (*n*, *k*) (for internal atoms we take *M*_*α*_ = *M*_0_, whereas for the edge atoms we take *M*_*α*_ = *M*_1_), **u**_*α*_ = [*u*_*α*,1_(*t*), *u*_*α*,2_(*t*), *u*_*α*,3_(*t*)] is the position vector of the carbon atom with the index *α* at the moment *t*. The term *P*_*α*_ describes the interaction of the atom with the index *α* = (*n*, *k*) and its neighboring atoms. The potential depends on variations in bond length, bond angles, and dihedral angles between the planes formed by three neighboring carbon atoms and it can be written in the form

18 $$P=\sum _{{{\rm{\Omega }}}_{1}}{U}_{1}+\sum _{{{\rm{\Omega }}}_{2}}{U}_{2}+\sum _{{{\rm{\Omega }}}_{3}}{U}_{3}+\sum _{{{\rm{\Omega }}}_{4}}{U}_{4}+\sum _{{{\rm{\Omega }}}_{5}}{U}_{5},$$

where Ω_*i*_, with *i* = 1, 2, 3, 4, 5, are the sets of configurations including all interactions of neighbors. This sets only need to contain configurations of the atoms shown in Fig. 15, including their rotated and mirrored versions.

The potential *U*_1_(**u**_*α*_, **u**_*β*_) describes the deformation energy due to a direct interaction between pairs of atoms with the indexes *α* and *β*, as shown in Fig. 15(a). The potential *U*_2_(**u**_*α*_, **u**_*β*_, **u**_*γ*_) describes the deformation energy of the angle between the valence bonds **u**_*α*_ **u**_*β*_ and **u**_*β*_ **u**_*γ*_, see Fig. 15(b). Potentials *U*_*i*_(**u**_*α*_, **u**_*β*_, **u**_*γ*_, **u**_*δ*_), *i* = 3, 4, and 5, describes the deformation energy associated with a change in the angle between the planes **u**_*α*_, **u**_*β*_, **u**_*γ*_ and **u**_*β*_, **u**_*γ*_, **u**_*δ*_, as shown in Fig. 15(c–e).

We use the potentials employed in the modeling of the dynamics of large polymer macromolecules^26, 27^: for the valence bond coupling,

19 $${U}_{1}({{\bf{u}}}_{1},{{\bf{u}}}_{2})={\varepsilon }_{1}{\{\exp [-{\alpha }_{0}(\rho -{\rho }_{0})]-1\}}^{2},\rho =|{{\bf{u}}}_{2}-{{\bf{u}}}_{1}|,$$

where *ε*_1_ = 4.9632 eV is the energy of the valence bond and *ρ*_0_ = 1.418 Å is the equilibrium length of the bond; the potential of the valence angle

20 $$\begin{array}{rcl}{U}_{2}({{\bf{u}}}_{1},{{\bf{u}}}_{2},{{\bf{u}}}_{3}) & = & {\varepsilon }_{2}{(\cos \phi -\cos {\phi }_{0})}^{2},\\ \cos \,\phi  & = & ({{\bf{u}}}_{3}-{{\bf{u}}}_{2},{{\bf{u}}}_{1}-{{\bf{u}}}_{2})/(|{{\bf{u}}}_{3}-{{\bf{u}}}_{2}|\cdot |{{\bf{u}}}_{2}-{{\bf{u}}}_{1}|),\end{array}$$

so that the equilibrium value of the angle is defined as $\cos \,{\phi }_{0}=\,\cos \,\mathrm{(2}\pi \mathrm{/3)=}-\mathrm{1/2}$; the potential of the torsion angle

21 $$\begin{array}{rcl}{U}_{i}({{\bf{u}}}_{1},{{\bf{u}}}_{2},{{\bf{u}}}_{3},{{\bf{u}}}_{4}) & = & {\varepsilon }_{i}(1+{z}_{i}\,\cos \,\varphi ),\\ \cos \,\varphi  & = & ({{\bf{v}}}_{1},{{\bf{v}}}_{2})/(|{{\bf{v}}}_{1}|\cdot |{{\bf{v}}}_{2}|),\\ {{\bf{v}}}_{1} & = & ({{\bf{u}}}_{2}-{{\bf{u}}}_{1})\times ({{\bf{u}}}_{3}-{{\bf{u}}}_{2}),\\ {{\bf{v}}}_{2} & = & ({{\bf{u}}}_{3}-{{\bf{u}}}_{2})\times ({{\bf{u}}}_{3}-{{\bf{u}}}_{4}),\end{array}$$

where the sign *z*_*i*_ = 1 for the indices *i* = 3, 4 (equilibrium value of the torsional angle *ϕ*_0_ = *π*) and *z*_*i*_ = −1 for the index *i* = 5 (*ϕ*_0_ = 0).

The specific values of the parameters are *α*_0_ = 1.7889 Å^−1^, *ε*_2_ = 1.3143 eV, and *ε*_3_ = 0.499 eV, and they are found from the frequency spectrum of small-amplitude oscillations of a sheet of graphite^28^. According to the results of ref. 29 the energy *ε*_4_ is close to the energy *ε*_3_, whereas ${\varepsilon }_{5}\ll {\varepsilon }_{4}$ (|*ε*_5_/*ε*_4_| < 1/20). Therefore, in what follows we use the values *ε*_4_ = *ε*_3_ = 0.499 eV and assume *ε*_5_ = 0, the latter means that we omit the last term in the sum (18).

The length of one elementary cell is $a=2{\rho }_{0}[1+\,\cos (\pi /3)]=3{\rho }_{0}=4.254$ Å. The length of the nanoribbon composed on *N* cells is *L* = *aN* + *ρ*_0_/2, and its width $D=2K{\rho }_{0}\,\cos (\pi /6)=K\sqrt{3}{\rho }_{0}$. For number of hexagons in the elementary cell *K* = 5, 7, 9, 13 and 21 nanoribbon width *D* = 1.23, 17.2, 2.21 3.19 and 5.15 nm.

More detailed discussion and motivation of our choice of the interaction potentials (19), (20), (21) can be found in ref. 23. Such potentials have been employed for modeling of thermal conductivity of carbon nanotubes^30^, graphene nanoribbons^23^ and also in the analysis of their oscillatory modes^31^ and localized defect modes in graphene^21^.

### Dispersion curves

In the equilibrium state, ${\{{{\bf{u}}}_{({\bf{n}},{\bf{k}})}\}}_{n=-\infty ,k=1}^{+\infty ,{N}_{e}}$, all atoms of ideal infinite nanoribbon are in the plane, all valent bonds have the equilibrium length *ρ* = *ρ*_0_ and all valent angle also have equilibrium value *ϕ* = 2*π*/3. We introduce 3*N*_*e*_-dimensional vector,

$${{\bf{x}}}_{n}=({{\bf{u}}}_{(n\mathrm{,1)}}-{{\bf{u}}}_{(n\mathrm{,1)}}^{0},\ldots ,{{\bf{u}}}_{(n,{N}_{e})}-{{\bf{u}}}_{(n,{N}_{e})}^{0}),$$

describing a shift of the atom of the *n*th cell from its equilibrium position. Then, the armchair nanoribbon Hamiltonian can be written in the following form:

22 $$H=\sum _{n}\{\frac{1}{2}({\bf{M}}{\dot{{\bf{x}}}}_{n},{\dot{{\bf{x}}}}_{n})+{\mathscr{P}}({{\bf{x}}}_{n},{{\bf{x}}}_{n+1})\},$$

where **M** is the diagonal matrix of masses of all atoms of the elementary cell, ${\mathscr{P}}({{\bf{x}}}_{n},{{\bf{x}}}_{n+1})$ is the interaction energy for the interaction between the cells with the numbers *n* and *n* + 1, that is a sum of the energies of all types of deformations.

Hamiltonian (22) generates the following set of the equations of motion:

23 $$-{\bf{M}}{\ddot{{\bf{x}}}}_{n}={{\bf{B}}}_{1}{{\bf{x}}}_{n}+{{\bf{B}}}_{2}{{\bf{x}}}_{n+1}+{{\bf{B}}}_{2}^{\ast }{{\bf{x}}}_{n-1},$$

where the matrix elements are defined as

$${{\bf{B}}}_{1}={{\mathscr{P}}}_{{{\bf{x}}}_{1},{{\bf{x}}}_{1}}+{{\mathscr{P}}}_{{{\bf{x}}}_{2},{{\bf{x}}}_{2}},\,\,{{\bf{B}}}_{2}={{\mathscr{P}}}_{{{\bf{x}}}_{1},{{\bf{x}}}_{2}},$$

and the matrix of the partial derivatives takes the form

$${{\mathscr{P}}}_{{{\bf{x}}}_{i},{{\bf{x}}}_{j}}=\frac{{\partial }^{2}{\mathscr{P}}}{\partial {{\bf{x}}}_{i}\partial {{\bf{x}}}_{j}}({\bf{0}},{\bf{0),\ \ }}i,j=\mathrm{1,2.}$$

Solutions of the system of linear Eq. (23) can be sought in the standard form

24 $${{\bf{x}}}_{n}=A{\bf{v}}\,\exp (iqn-i\omega t),$$

where *A* is the mode amplitude, *q* ∈ [0, *π*] is the dimensional wave number, and *ω* is the phonon frequency. Substituting expression (24) into the system (23), we obtain the eigenvalue problem

25 $${\omega }^{2}{\bf{Mv}}=[{{\bf{B}}}_{1}+{{\bf{B}}}_{2}{e}^{iq}+{{\bf{B}}}_{2}^{\ast }{e}^{-iq}]{\bf{v}}\mathrm{.}$$

The problem (25) can be rewritten in the form

26 $${\omega }^{2}{\bf{e}}={{\bf{M}}}^{-\mathrm{1/2}}[{{\bf{B}}}_{1}+{{\bf{B}}}_{2}{e}^{iq}+{{\bf{B}}}_{2}^{\ast }{e}^{-iq}]{{\bf{M}}}^{-\mathrm{1/2}}{\bf{e}},$$

where the vector **v** = **M** ^−1/2^ **e**, **e** is the normalized dimensionless vector [(**e**, **e**) = 1].

Therefore, in order to find the dispersion relations characterizing the modes of the nanoribbon for each fixed value of the wave number 0 ≤ *q* ≤ *π* we need to find the eigenvalues of the Hermitian matrix (26) of the order 3*N*_*e*_ × 3*N*_*e*_. As a result, the dispersion curves are composed of 3*N*_*e*_ branches ${\{{\omega }_{j}(q)\}}_{j=1}^{3{N}_{e}}$. Two third of the branches corresponds to the atom vibrations in the plane of the nanoribbon *xy* (in-plane vibrations), whereas only one third corresponds to the vibrations orthogonal to the plane(out-of-plane vibrations), when the atoms are shifted along the axes *z*.

As is seen in Figs 7 and 8, the spectrum of the in-plane nanoribbon oscillations occupies the frequency interval [0, *ω*_*i*_] and the spectrum of the out-of-plane oscillations – interval [0, *ω*_*o*_], where maximum (cutoff) frequencies *ω*_*i*_ = 1600, *ω*_*o*_ = 900 cm^−1^. This values agrees well with the experimental data for a planar graphite^32, 33^.

#### Local defects in graphene

First, we introduce a model of a graphene nanoribbon with defects. We take a finite-size armchair nanoribbon with an ideal honeycomb lattice (see Fig. 14) and, depending on the type of defect, we add or remove some carbon atoms at its center by cutting the corresponding chemical bonds. In this way, we define the atomic configurations ${\{{{\bf{u}}}_{(n,k)}^{0}\}}_{n=1,k=1}^{N,{N}_{e}}$ that will relax to a stationary structure with a specific type of defect. To find the ground state of the nanoribbon with defects, we should find the energy minimum for the interaction energy,

27 $$E=\sum _{n=1}^{N}\sum _{k=1}^{{N}_{e}}{P}_{(n,k)}\to \,{\rm{\min }}\,:{\{{{\bf{u}}}_{(n,k)}\}}_{n=1,k=1}^{N,{N}_{e}},$$

where term *P*_(*n*,*k*)_ describes the interaction of the carbon atom with index (*n*, *k*) and its neighboring atoms (for detail see ref. 21).

We consider the armchair graphene nanoribbon with length *L* = *Na* + *ρ*_0_/2 = 86 nm (number of elementary cells *N* = 203) and different widths, namely *D* = 1.23, 1.72, 2.21, 3.19 nm (the number of atoms in the elementary cell is *N*_*e*_ = 22, 30, 38, and 54, respectively). A local defect is placed in the center of the nanoribbon.

We consider *seven types* of local defects that are most common in the graphene lattices^11^ and place them at the center of the nanoribbon – see Fig. 2. The defects are: the local edge defects ED_+2_ (two extra carbon atoms which create one extra hexagon at the edge), ED_−2_ (two missing carbon atoms and one missing hexagon), see Fig. 2(a,b); central double vacancy V_2_(585), as shown in Fig. 2(c); central Stone-Wales defect SW(5577) as shown in Fig. 2(d); isolated central vacancy V_1_(59) (missing one carbon atoms) shown in Fig. 2(e), and the inverse Stone-Wales defect I_2_(7557) when two extra carbon atoms are added to the lattice, see Fig. 2(f). In addition, we consider a transverse array of the inverse Stone-Walles defects, as shown in Fig. 2(g).

In order to find the ground state of the system with defect, the problem (27) is solved numerically by means of the conjugate gradient method. If ${\{{{\bf{u}}}_{(n,k)}^{0}\}}_{n=1,k=1}^{N,{N}_{e}}$ is the ground state of the nanoribbon with defect, then for small-amplitude oscillations we can write ${{\bf{u}}}_{(n,k)}(t)={{\bf{u}}}_{(n,k)}^{0}+{{\bf{v}}}_{(n,k)}(t)$, where $|{{\bf{v}}}_{(n,k)}|\ll {\rho }_{0}$. Then, the equations of motion corresponding to the Hamiltonian (17) can be written as system of 3*N*_*a*_ linear equations for 3*N*_*a*_ variables,

28 $$-{\bf{M}}{\ddot{{\bf{v}}}}_{j}=\frac{\partial H}{\partial {{\bf{u}}}_{j}}=\sum _{i=1}^{{N}_{a}}{B}_{ij}{{\bf{v}}}_{i},$$

where 3*N*_*a*_ × 3*N*_*a*_ matrix

29 $${B}_{ij}={\frac{{\partial }^{2}H}{\partial {{\bf{u}}}_{j}\partial {{\bf{u}}}_{i}}|}_{{\{{{\bf{u}}}_{l}^{0}\}}_{l=1}^{{N}_{a}}}\mathrm{.}$$

For convenience, here we used a direct numeration of atoms of the nanoribbon with one index only, *N*_*a*_ = *NN*_*e*_ + (*K* − 1)2 − 1 is the number of atoms, and ${\bf{M}}={\{{M}_{j}{\delta }_{ij}\}}_{i=1,j=1}^{3{N}_{a},3{N}_{a}}$ is the diagonal matrix of the carbon masses and edge CH groups.

To find all linear modes of the nanoribbon, we need to find numerically all 3*N*_*a*_ eigenvalues and corresponding eigenvectors of the eigenvalue problem

30 $$\lambda {\bf{Mv}}={\bf{Bv}},$$

where *B* = (*B*_*i*,*j*_) is real symmetric matrix. The problem (30) can be rewritten in the form

31 $$\lambda {\bf{e}}={{\bf{M}}}^{-\mathrm{1/2}}{\bf{B}}{{\bf{M}}}^{-\mathrm{1/2}}{\bf{e}},$$

where the vector **v** = **M** ^−1/2^ **e**.

If we define *λ* and ${\bf{e}}={\{{{\bf{e}}}_{j}^{0}\}}_{j=1}^{{N}_{a}}$ as the eigenvalue and normalized eigenvector of the problem (31), (**e**, **e**) = 1, then solution of Eq. (28) will have the form ${{\bf{v}}}_{j}(t)=A{{\bf{v}}}_{j}^{0}\exp (i\omega t)$, where ${{\bf{v}}}_{j}^{0}={{\bf{e}}}_{j}^{0}/{M}_{j}^{\mathrm{1/2}}$, mode frequency $w=\sqrt{\lambda }$ and *A* is the mode amplitude.

### Localized vibrational modes supported by defects

Since a nanoribbon in the ground state is a planar object, we divide its vibrational modes into two classes: the planar modes when atoms move in the two-dimensional space, and the out-of-plane modes when the atoms move perpendicular to the planar structures. To reduce the dimension of the corresponding matrices in the problem (30), such vibrational modes are useful to treat separately. We notice that such a variable separation is valid only in the small-amplitude approximation when the motion equations (28) become linear.

First, we analyze eigenvectors and find the modes localized on defects. We characterize the degree of spatial localization of the oscillatory eigenmode by the parameter of localization (inverse participation number), $d={\sum }_{j=1}^{{N}_{a}}{({{\bf{e}}}_{j}^{0},{{\bf{e}}}_{j}^{0})}^{2}={\sum }_{j=1}^{{N}_{a}}{M}_{j}^{2}{({{\bf{v}}}_{j}^{0},{{\bf{v}}}_{j}^{0})}^{2}$. For the modes which are not localized in space, *d* ≈ 1/*N*_*a*_, for the mode localized on single atom, *d* = 1. Inverse value *N*_*d*_ = 1/*d* (participation number) characterizes the number of atoms which are involved into this oscillatory mode. We define the eigenmode as being spatially localized if its participation number *N*_*d*_ < 200 (*d* > 0.005), meaning that the vibrational state is localized on less than 200 atoms.

**Figure 16:**
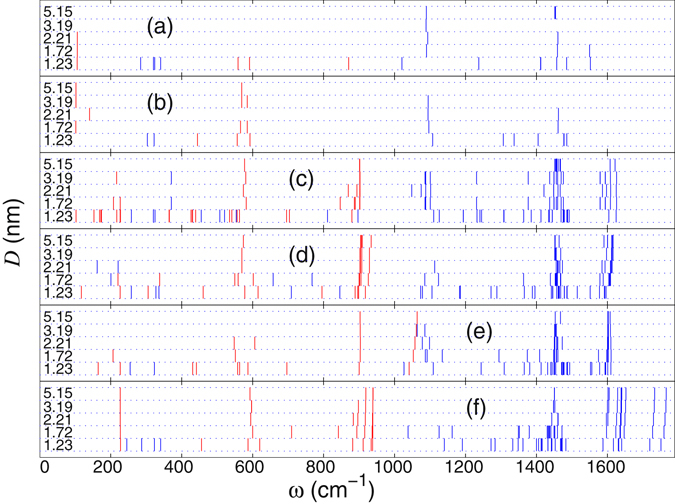
Eigenfrequency spectrum of the vibrational local models supported by the edge defects ED_+2_ (**a**), ED_−2_ (**b**) and the central point defects V_2_(585) (**c**), SW(5577) (**d**), V_1_(59) (**e**), and I_2_(7557) (**f**). Nanoribbon has the width of *D* = 1.23, 1.72, 2.21, 3.19 and 5.15 nm, respectively. Red color marks the eigenfrequencies of out-of-plane vibrational modes, and blue color marks in-plane oscillatory modes.

Dependence of the eigenmode’s frequencies on the width of the graphene nanoribbon is summarized in Fig. 16, for six types of defects discussed above. As follows from those results, for all types of defects we observe the general trend that the number of localized modes is decreasing when the width of the nanoribbon is increasing. For the maximum studied width *D* = 5.15 nm the localized modes supported by the internal defects V_2_, SW(5577), V_1_(59), and I_2_(7557) are not affected by the edges of the nanoribbon, so their frequencies coincide with those in an infinite graphene sheet. In narrow nanoribbons, we observe the appearance of additional localized modes in the vicinity of the defects. Similar conclusions are valid for the edge defects ED_±2_ which in general have a lower number of localized modes. Thus, resonant effects in the phonon scattering should be expected to be more pronounced for narrow nanoribbons and for internal defect states.
